# Editorial: Nanostructured functional materials for smart window, anti-corrosion/fouling and electronic packaging

**DOI:** 10.3389/fchem.2022.1118210

**Published:** 2022-12-20

**Authors:** Ning Wang, Lin Gu, Yujie Ke, Ying Zhong

**Affiliations:** ^1^ Shenzhen Institute of Advanced Electronic Materials, Shenzhen Institute of Advanced Technology, Chinese Academy of Sciences, Shenzhen, China; ^2^ School of Chemical Engineering and Technology, Sun Yat-sen University, Zhuhai, China; ^3^ NanoBio Lab, Institute of Materials Research and Engineering, Agency for Science, Technology and Research (A*STAR), Singapore, Singapore; ^4^ Engineering Product Development, Singapore University of Technology and Design, Singapore, Singapore; ^5^ School of Materials Science and Engineering, Harbin Institute of Technology, Shenzhen, China

**Keywords:** nanomaterials, functional materials, anticorrosion, antifouling, energy materials

It is our pleasure to organize the Research Topic “*Nanostructured Functional Materials for Smart Window, Anti-corrosion/fouling and Electronic Packaging*” that was proposed to present the recent advances in the functional nanomaterials for the smart applications relevant to green energy, corrosion/fouling resistance as well as the information technology.

To meet the CO_2_ reduction requirement for saving our humankind on the Earth, three approaches have been developed based on materials science and engineering. The first approach is the green energy applications, e.g., smart window, fuel cells and photovoltaics that could substitute the traditional fossil fuels. The second way is to extend the lifespan of the existed consumables though the anticorrosion/antifouling post-treatment. The last but not the least is to develop the high-durability and high-efficient information technology *via* the advanced electronic packaging that could enormously reduce the energy consumption *via* the smart automation. Therefore, the research subjects in this Research Topic may be able to inspire new ideas for chemists in protecting our environment. In this Research Topic, we collected 4 papers, which will be introduced subsequently.

Meng et al., reported a solid-state mixing method to prepare a Pr_0.8_Sr_0.2_Fe_0.7_Ni_0.3_O_3−δ_-Pr_1.2_Sr_0.8_Fe_0.4_Ni_0.6_O_4+δ_ (PSFN_113-214_) composite cathode oxide for the solid oxide fuel cells (SOFCs). In this work, the oxygen vacancy content could be increased by mixing the PSFN_214_ and PSFN_113_, where a heterostructure was formed and resulted in the promotion of oxygen ion transport as well as the specific surface area. The optimum mixing ratio 5:5 gave rise to the highest oxygen vacancy content and the largest specific surface area, and thus led to the strongest interface effect. The corresponding maximum power density was .699 W cm^−2^, which was nearly 1.44 times of PSFN_113_ and 1.24 times of PSFN_214_. This new PSFN_113-214_ composite may be an alternative cathode oxide for SOFCs.

Liang et al., investigate the effects of MgO and Fe_2_O_3_ dual sintering aids on the microstructure and electrochemical properties of solid state Gd_0.2_Ce_0.8_O_2-δ_ (GDC) electrolytes for the SOFCs. The addition of MgO and Fe_2_O_3_ was found to be able to reduce the sintering temperature, increase densification and decrease the grain boundary resistance of the electrolyte. The optimun 2 mol% MgO and 2 mol% Fe_2_O_3_ co-doped GDC (GDC-MF) exhibits the highest grain boundary conductivity. The grain boundary conductivity and total conductivity of GDC-MF at 400°C are 15.89 and 5.56 times larger than pure GDC, respectively that generated a 47% higher ORR efficiency and 36.7% larger single-cell peak power density. The newly designed electrolyte should be promising for the intermediate-temperature solid oxide fuel cells (IT-SOFCs).

Zhang et al., reported a dispersible graphene-based material with aggregation-induced emission (AIE) effect prepared by a wet chemical reduction method. In the wet method, a conjugated molecule TPEP containing tetraphenylethylene (TPE) and pyrene with π–π interactions and a wrapping effect was employed as a stabilizer. The rGO-TPEP showed a AIE effect and a good dispersibility that resulted in 2.23 times higher fluorescence intensity than TPEP. In the aggregated state, the trace 2,4-dinitrotoluene (DNT) can be detected by the rGO-TPEP even at .91 ppm concentration, and the quenching constant could reach 2.47 × 104 M^−1^.

Zhang et al., reported a mussel-inspired dopamine-modified sodium alginate (SA-DA) and the application as antibacterial coatings on cotton fabric, aluminum sheet, and polyurethane membrane. The coatings were constructed through layer-by-layer deposition of polyhexamethylene guanidine and sodium alginate. The coated cotton fabric exhibited ideal hydrophilicity, and the liquid absorption capacity increased with the coating layers. The growth of *Escherichia coli* and *Staphylococcus aureus* was notably inhibited on the coated cotton fabric, and 10 coating bilayers achieved 100% inhibition of bacterial growth within 10 min. In addition, the coated cotton fabric could promote blood clotting by concentrating the components of blood and activating the platelets, and no significant hemolysis and cytotoxicity could be observed. The coated aluminum and polyurethane film also displayed an obvious antibacterial effect. This work proposed an alternative approach for designing the antibacterial coating tactics for substrates.

